# Multiple drug resistance protein (*MDR-1*), multidrug resistancerelated protein (*MRP*) and lung resistance protein (LRP) gene expression in childhood acute lymphoblastic leukemia

**DOI:** 10.1590/S1516-31802004000400007

**Published:** 2004-07-01

**Authors:** Elvis Terci Valera, Carlos Alberto Scrideli, Rosane Gomes de Paula Queiroz, Bianca Maria Ortelli Mori, Luiz Gonzaga Tone

**Keywords:** Drug resistance, Cancer, Children, Leukemia, Acute lymphocytic leukemia, Genes, Resistência a drogas, Câncer, Criança, Leucemia, Leucemia linfoblástica aguda, Genes

## Abstract

**CONTEXT:**

Despite the advances in the cure rate for acute lymphoblastic leukemia, approximately 25% of affected children suffer relapses. Expression of genes for the multiple drug resistance protein (*MDR-1*), multidrug resistance-related protein (*MRP*), and lung resistance protein (*LRP*) may confer the phenotype of resistance to the treatment of neoplasias.

**OBJECTIVE:**

To analyze the expression of the *MDR-1*, *MRP* and *LRP* genes in children with a diagnosis of acute lymphoblastic leukemia via the semiquantitative reverse transcription polymerase chain reaction (RT-PCR), and to determine the correlation between expression and event-free survival and clinical and laboratory variables.

**DESIGN:**

A retrospective clinical study.

**SETTING:**

Laboratory of Pediatric Oncology, Department of Pediatrics, Faculdade de Medicina de Ribeirão Preto, Universidade de São Paulo, Brazil.

**METHODS:**

Bone marrow aspirates from 30 children with a diagnosis of acute lymphoblastic leukemia were assessed for the expression of messenger RNA for the *MDR-1*, *MRP* and *LRP* genes by semi-quantitative RT-PCR.

**RESULTS:**

In the three groups studied, only the increased expression of *LRP* was related to worsened event-free survival (p = 0.005). The presence of the common acute lymphoblastic leukemia antigen (CALLA) was correlated with increased *LRP* expression (p = 0.009) and increased risk of relapse or death (p = 0.05). The relative risk of relapse or death was six times higher among children with high *LRP* expression upon diagnosis (p = 0.05), as confirmed by multivariate analysis of the three genes studied (p = 0.035).

**DISCUSSION:**

Cell resistance to drugs is a determinant of the response to chemotherapy and its detection via RT-PCR may be of clinical importance.

**CONCLUSIONS:**

Evaluation of the expression of genes for resistance to antineoplastic drugs in childhood acute lymphoblastic leukemia upon diagnosis, and particularly the expression of the *LRP* gene, may be of clinical relevance, and should be the object of prospective studies.

## RESUMO

**CONTEXTO:**

Apesar dos avanços nos índices de cura da leucemia linfoblástica aguda (LLA) aproximadamente 25% das crianças sofrem recaídas da doença. A expressão dos genes de resistência múltipla a drogas (*MDR-1*), genes relacionados à proteína de resistência múltipla a drogas (*MRP*) e genes da proteína de resistência pulmonar (*LRP*) podem conferir o fenótipo de resistência ao tratamento das neoplasias.

**OBJETIVO:**

Analisar a expressão dos genes de resistência *MDR-1*, *MRP* e *LRP* em crianças diagnosticadas com LLA por meio da técnica da reação em cadeia da polimerase da transcriptase reversa (RT-PCR) semiquantitativa, associando estas expressões à sobrevida livre de eventos (SLE) e a variáveis clínico-laboratoriais.

**TIPO DE ESTUDO:**

Estudo clínico retrospectivo.

**LOCAL:**

Laboratório de Oncologia Pediátrica do Departamento de Puericultura e Pediatria da Faculdade de Medicina de Ribeirão Preto — Universidade de São Paulo, Brasil.

**MÉTODOS:**

Amostras de medula óssea de 30 crianças com o diagnóstico de leucemia linfoblástica aguda foram avaliadas quanto à expressão do RNA-mensageiro para os genes *MDR-1*, *MRP* e *LRP*, pela reação em cadeia da RT-PCR semiquantitativa.

**RESULTADOS:**

Dos três genes estudados, somente a expressão aumentada de *LRP* esteve relacionada a uma pior SLE (p = 0.005). A presença do antígeno para leucemia linfoblástica aguda comum (CALLA) se correlacionou à expressão aumentada de *LRP* (p = 0.009) e a risco aumentado de ocorrência de recaída ou óbito (p = 0.05). O risco relativo de ocorrência de recaída ou óbito é seis vezes maior em crianças com alta expressão de *LRP* ao diagnóstico (p = 0.05), o que se confirma na análise multivariada dos três genes estudados (p = 0.035).

**DISCUSSÃO:**

A resistência celular a drogas é um determinante de resposta ao tratamento oncológico e sua avaliação por RT-PCR pode ser de importância.

**CONCLUSÕES:**

A avaliação da expressão dos genes de resistência a drogas antineoplásicas na leucemia linfoblástica aguda da criança ao diagnóstico, particularmente do gene *LRP*, pode ser de relevância clínica e deve ser objeto de estudos prospectivos.

## INTRODUCTION

Despite the great advances in the cure rate for acute lymphoblastic leukemia (ALL), approximately 25% of affected children present disease recurrence.^1^ Treatment failure can be explained in part by pharmacokinetic mechanisms that reduce the time length or effective level of leukemic blast exposure to the drug, also known as pharmacokinetic resistance. It can also be partially explained by cell resistance to drugs.^2^ Cell resistance to antineoplastic drugs is seen as one of the most significant barriers against effective treatment of malignant tumors in general.^3^

The description of the multiple drug resistance protein (*MDR-1*), also called pglycoprotein, seemed to be promising for the understanding of the mechanisms of antineo-plastic treatment failure.^4^ Subsequently, other drug resistance genes were described, among them genes related to resistance (multidrug resistance-related protein, *MRP*) and genes for the lung resistance protein (*LRP*). However, it appears that none of these can fully explain the pathways that effectively lead to the occurrence of tumor resistance.

The situation regarding the resistance to treatment presented by some forms of hemato-logical malignancies such as acute lymphoblastic leukemia in children is still controversial.^5^ The relationships between *MDR-1*, *MRP* and *LRP* expression, resistance to treatment and survival among children with acute lymphoblastic leukemia are still unclear.^5,6^ The incorporation of new knowledge about the mechanisms of tumor resistance to antineoplastic drugs may contribute towards increasing the chances of cure, either through the development of new drugs, or by means of strategies that may modulate or reverse the resistance.^7^

The objective of the present study was to semi-quantitatively analyze the expression of the resistance genes *MDR-1*, *MRP* and *LRP* in children with acute lymphoblastic leukemia, and to correlate such expressions with event-free survival and clinical and laboratory variables.

## METHODS

### Patients

In this retrospective clinical study, 30 patients (16 males and 14 females) with a diagnosis of acute lymphoblastic leukemia, who were admitted for treatment to the Department of Pediatrics of the University Hospital, Faculdade de Medicina de Ribeirão Preto, Universidade de São Paulo, from June 1998 to June 2003, were assessed for the expression of messenger RNA (mRNA) for *MDR-1*, *MRP* and *LRP*, after their parents or guardians gave informed consent. Median patient age was 59 months (range: 5-156 months).

Seventeen patients had a peripheral leukocyte count of less than 50,000/mm^3^ upon diagnosis. The patients were classified according to immunophenotype as precursor B-ALL (3 cases), common ALL-B (18 cases), mature ALL-B (2 cases), and ALL-T (7 cases). The patients were classified as being at low risk (11 cases) or at high risk (19 cases) of suffering a relapse according to the norms of the Brazilian Group for Treatment of Childhood Leukemia (BGTCL).^8^ Patients were defined as being at low risk when they were 1 to 9 years old and when their leukocyte count was less than 50,000/mm^3^; all other patients were defined as high risk. Two patients were analyzed upon diagnosis and relapse. The diagnosis of leukemia was based on bone marrow cytology and on immunological investigation with monoclonal antibodies using flow cytometry. Minimal residual disease (MRD) was determined by the polymerase chain reaction (PCR) for rearrangements of T-cell receptors (TCR) and the immunoglobulin heavy chain (IgH), with consensus primers at the end of induction for acute lymphoblastic leukemia. The patients were treated according to the 1993 and 1999 BGTCL protocols.^8^

### RNA extraction

The material (0.5 to 1 ml of bone marrow aspirate) was collected into a tube containing ethylenediaminotetraacetic acid (EDTA), chilled on ice and immediately processed for RNA extraction using the TRIzol^®^LS reagent kit (Invitrogen, Life Technologies) according to the manufacturer's instructions. The integrity of the RNA obtained was assessed by agarose gel electrophoresis of RNA and by RT-PCR (reverse transcription PCR) for the housekeeping gene of beta-globin. Only samples with RNA of satisfactory quality were included in the study.

### RT -PCR assay

For semiquantitative RT-PCR, a total of 0.2 μg RNA was used in the reverse transcription reactions to obtain complementary DNA (cDNA). The resulting cDNA was quantified by spectrophotometry at an optical density (OD) of 260, with the entire PCR procedure using about 1 μg cDNA per sample.

PCR was performed as follows: water for the reaction (18.05 μl) was added to 2.5 μl buffer, 0.75 μl magnesium chloride, 0.5 μl phosphate dinucleotide (DNTP set, 100 mM solutions; Amersham Pharmacia Biotech, NJ, USA) and 0.1 μl Taq-polymerase (Amersham Pharmacia Biotech). The primers for each of the genes under study were added to the reaction (0.5 μl in the forward and reverse forms).

The sequences of the primers used were: for *MDR-1*, 5'CCCATCATTGCAATAGCAGG (forward)/5'GTTCAAACTTCTGCTCCTGA (reverse); for *MRP*, 5'TGGGACTGGAATGTCACG (forward) 5'AGGAATATGCCCCGACTTC (reverse); for *LRP*; 5'GTCTTCGGGCCTGAGCTGGTGTCG (forward)/5'CTTGGCCGTCTCTTGGGGGTCCTT (reverse); and for beta-globin, 5'GGCAGAGCCATCTATTGCTTA (forward)/ 5'CCTTAGGGTTGCCCATAAC (reverse). The resulting amplification products consisted of 184, 260, 240 and 250 base pairs, respectively.

The PCR was standardized in a pilot study for the definition of the best denaturing, annealing and amplification conditions. The final PCR conditions were as follows: after the initial denaturation at 94° C for five minutes and pairing at 53° C for two minutes, each sample was submitted to 30 denaturation cycles at 94° C for one minute, pairing at 53° C for one minute, and extension at 72° C for one minute, followed by a final extension at 72° C for 10 minutes in a PTC-200 thermocycler (MJ Research, Inc, USA).

The PCR products were submitted to 2% agarose gel electrophoresis with ethidium bromide. Each patient was studied in triplicate. The images were photographed and filed for the study of band intensity. Band intensity was read for semi quantification using a computerized densitometer (GS 800 Calibrated Densitometer, BioRad, Hercules, CA, USA). The images were digitized and analyzed using reading software (Quantity One – Quantitation Software, BioRad). The values of the bands studied were normalized using the intensity of the marker band of molecular weight 300 base pairs (Invitrogen Tech-line, CA, USA).

### Control cell lines and positivity values

As the control for normal expression, we used the K562 cell line (resistance-free leukemic cells) and, as the positive control for *MDR-1*, we used the K562-LUCENA lineage, which is resistant to chemotherapy (anthracyclines and vinca alkaloids) due to the increase in *MDR-1* protein expression. Two distinct models for the cutoff of expression positivity were adopted. The first type of analysis (positivity criterion 1) considered gene expression above 50% of the normal expression value (expression of the non-drug-resistant K-562 leukemic lineage) to be positive and any expression below this cut-off line to be negative. The second type of analysis (positivity criterion 2) was used to divide the patients into groups with high and low expression levels depending on the median values detected for *MDR-1*, *MRP* and *LRP*. Patients with values below the median were considered to have low gene expression and those with values above the median were considered to have increased expression.

### Statistical analysis

Kaplan-Meier analysis was used to determine event-free survival and the various groups were compared by the log-rank and Breslow tests. The presence or relapse of the disease or the occurrence of death related to the disease were considered to be unfavorable events. The correlation of the *MDR-1*, *MRP* and *LRP* genes with age upon diagnosis, leukocyte count, race, immunophenotypic classification, French-American-British (FAB) classification^9^, presence of the marker for the common acute lymphoblastic leukemia antigen (CALLA), infiltration of the central nervous system (CNS), minimal residual disease on day 28 of induction, risk of relapse, and occurrence of relapse or death was calculated via the chi-squared or Fisher exact test (positivity cut-off 1) and via the nonparametric Mann-Whitney test (positivity cutoff 2). The relative risk (odds ratio) for the occurrence of an unfavorable event in the presence of increased *MDR-1*, *MRP* and *LRP* expression was calculated by the Fisher exact test. The level of significance was set at p < 0.05 for all analyses. The adjusted model of multivariate logistic regression analysis was applied to test the independence of the expression of the *MRD-1*, *MRP* and *LRP* genes.

## RESULTS

**Figure 1:**
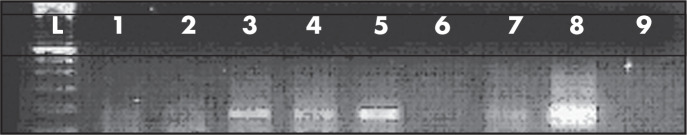
Photograph of the reverse transcriptase polymerase chain reaction agarose gel reaction for multiple drug resistance. L = molecular weight marker. Lanes 1 to 6: samples studied. Lane 7: K562 line. Lane 8: K562-LUCENA line. Lane 9: negative control.

Thirty bone marrow samples from children with acute lymphoblastic leukemia were collected upon diagnosis and two upon relapse. Figure 1 illustrates the agarose gel results of the cases studied. Lanes 1 to 9 refer to the study of *MDR-1* gene expression (bands with a molecular weight of 190 kd). Wide variations in band intensity among the sample studied can be seen (lanes 1 to 6). Lane 7 corresponds to the expression of the non-resistant K562 leukemic line. Lane 8 shows the strong intensity obtained with the drug-resistant K562-LUCENA line, through overexpression of the *MDR-1* gene. Lane 9 identifies the negative control for the reaction.

Analysis of positivity cut-off 1 showed that 40% of patients had increased *MDR-1* expression, 16.6% had increased *MRP* expression, and 33% had increased *LRP* expression upon diagnosis. Double positivity of the expression of drug resistance genes was observed in 26.6% of cases. Table 1 summarizes the event-free survival findings over a 48-month period for the patients with positive expression *MDR-1*, *MRP* and *LRP* genes and their respective levels of significance, and for patients without such expression. Only increased *LRP* expression upon diagnosis had a negative impact on survival (p = 0.005; log-rank test).

**Table 1:** Cumulative survival (%) over a period of 48 months for lymphoblastic leukemia patients with positive expression of the *MDR-1*, *MRP* and *LRP* genes and their respective levels of statistical significance, and for patients without such expression

		Cumulative survival (%)	log-rank	Breslow
MDR-1	+	59.66	ns (p = 0.600)	ns (p = 0.364)
		-	66.80	
MRP	+	63.07	ns (p = 0.229)	ns (p = 0.112)
		-	75.69	
LRP	+	25.00	p = 0.005*	p = 0.004*
		-	83.51	

*MDR-1 = multiple drug resistance-1 protein; MRP = multidrug resistance-related protein; LRP = lung resistance protein; ns = not significant.*

Similarly, the analysis of positivity based on the median values obtained (positivity cut-off 2) did not reveal any prognostic significance of *MDR-1* and *MRP* overexpression upon diagnosis (p = 0.344 and 0.695, respectively), whereas increased expression of *LRP* mRNA was associated with worsened event-free survival (p = 0.046). Absence of the expression of resistance genes or of one, two or more such genes had no adverse impact on event-free survival (p = 0.420, 0.009 and 0.260, respectively). The correlations between increased expression of the *MDR-1*, *MRP* and *LRP* genes and the following variables of clinical and laboratory importance in childhood acute lymphoblastic leukemia were also calculated: age upon diagnosis, leukocyte count, race, immunophenotypic classification, FAB classification, presence of the CALLA marker, infiltration of the central nervous system, minimal residual disease on day 28 of induction, risk of relapse, and occurrence of relapse or death (Table 2). It can be seen that high *LRP* expression upon diagnosis was associated with an increased risk of the occurrence of death and/or relapse (p = 0.05) and with higher occurrence of positivity for CALLA (p = 0.009). There was also a correlation between higher MRP expression and positivity for CALLA (p = 0.01).

**Table 2:** Median values, standard deviations and significance levels of the correlations between *MDR-1*, *MRP* and *LRP* and the remaining clinical-laboratory variables in 30 children with lymphoblastic leukemia

		MDR-1		MRP		LRP	
		P50 ± SD	p	P50 ± SD	p	P50 ± SD	p
	> 10,000	2.61 ± 5.70	0.63	0.83 ± 1.33	0.95	3.79 ± 9.11	0.66
LC	> 50,000	3.42 ± 6.8	0.81	1.12 ± 1.53	0.10	1.99 ± 2.65	0.54
	> 100,000	6.04 ± 10.29	0.15	1.48 ± 2.39	0.91	2.16 ± 2.33	0.48
	B	1.81 ± 3.06		0.93 ± 1.34		3.56 ± 8.91	
IP			0.44		0.70		0.56
	T	3.86 ± 9.05		0.39 ± 0.45		1.33 ± 2.12	
	Positive	0.51 ± 2.00		0.38 ± 0.49		0.40 ± 3.59	
CALLA			0.66		0.01		0.009
	Negative	1.27 ± 1.26		2.17 ± 2.03		3.49 ± 7.23	
	< 1 and > 10	4.55 ± 8.10		1.04 ± 1.91		2.47 ± 3.43	
Age	1 - 10	1.47 ± 3.13	0.07	0.71 ± 0.87	0.63	3.25 ± 9.03	0.66
	White	2.75 ± 6.14		0.69 ± 1.25		3.72 ± 9.71	
Race			0.64		0.53		0.74
	Non-white	1.49 ± 1.80		1.00 ± 1.16		1.87 ± 2.81	
	Positive	2.47 ± 5.23		0.75 ± 1.26		3.24 ± 8.29	
Cerebrospinal fluid	Negative	0.71 ± 0.57	0.95	1.25 ± 0.49	0.06	1.22 ± 0.78	0.74
	L1	1.99 ± 3.58		0.95 ± 1.57		1.32 ± 1.87	
FAB			0.77		0.87		0.24
	L2	2.88 ± 6.87		0.54 ± 0.56		5.70 ± 12.0	
	Positive	4.17 ± 8.93		0.66 ± 0.62		7.74 ± 15.6	
MRD			0.61		0.78		0.19
	Negative	1.79 ± 3.34		0.95 ± 1.14		1.73 ± 2.56	
	Low	1.86 ± 3.31		0.42 ± 0.68		1.22 ± 0.78	
Risk of relapse	High	2.94 ± 6.91	0.82	1.06 ± 1.42	0.06	5.00 ± 12.1	0.98
	Favorable	2.77 ± 6.04		0.46 ± 0.61		1.50 ± 2.58	
Event			0.40		0.07		0.05
	Unfavorable	1.33 ± 1.28		1.48 ± 1.78		6.13 ± 13.0	

*MDR-1 = multiple drug resistance-1 protein; MRP = multidrug resistance-related protein; LRP = lung resistance protein; P50 = 50th percentile; SD = standard deviation; p = level of statistical significance; LC = leukocytes (mm3); IP = immunophenotype; CALLA = common acute lymphoblastic leukemia antigen; FAB = French-American-British classification; MRD = minimal residual disease on day 28 of induction.*

Univariate analysis showed a correlation between the occurrence of an unfavorable event, characterized as relapse and/or death due to the disease, and positive expression of the *LRP* gene. The relative risk (odds ratio) of the occurrence of an unfavorable event was six times higher for patients who were *LRP* positive upon diagnosis (p = 0.050; Mann-Whitney test). The adjusted model for multivariate logistic analysis was impaired by the reduced sample size. However, it was only possible to perform multivariate analysis for the three genes as a whole, without considering other variables. The results are presented in Table 3. It can be seen that, once again, only the positive expression of the *LRP* gene upon diagnosis had an adverse impact on the clinical course (p = 0.035).

**Table 3:** Multivariate analysis, coefficients, standard errors and significance levels for the correlations between positivity of the *MDR-1*, *MRP* and *LRP* genes upon diagnosis and risk of death and/or disease recurrence in 30 children with lymphoblastic leukemia.

	Coefficient	Standard error	p
**MDR-1**	0.70	0.919	p = 0.661
**MRP**	1.68	1,32	p = 0.485
**LRP**	1.79	0.85	p = 0.035

*MDR-1 = multiple drug resistance-1 protein; MRP = multidrug resistance-related protein; LRP = pulmonary resistance protein.*

### Relapse

Of the five documented cases of relapse, it was only possible to assess the two for which RNA samples of satisfactory quality were available. Case 1 was a white girl with a diagnosis of leukemia made at 19 months of life. She presented acute lymphoblastic leukemia of pre-B immunophenotype, positive CALLA and 73,500 leukocytes/mm^3^ upon diagnosis. She died within six months due to disease that was refractory to treatment. This child had low expression of the three genes studied upon diagnosis and upon relapse, she started to present increased expression of the *MDR-1* gene (1.53), even though this value was borderline for the stipulated positivity cutoff.

Case 2 was diagnosed as acute lymphoblastic leukemia at 43 months of life, with pre-B immunophenotype, positive CALLA and 20,300 leukocytes/mm^3^ upon admission. He suffered medullary relapse during the 29th week of treatment. The drug resistance data revealed increased expression of the *MDR-1* gene (5.10) upon diagnosis, with initially normal expression of the *MRP* and *LRP* genes (0.38 and 0.43, respectively). Upon relapse, there was an inversion of these findings, with absence of positivity of the *MDR-1* gene and high expression of the *MRP* and *LRP* genes (3.92 and 4.57, respectively).

## DISCUSSION

Although the antineoplastic drugs currently available are usually effective for the treatment of various tumors, they may prove to be relatively ineffective in the treatment of some primary or recurrent neoplasias. The identification of factors that might effectively predict the response of the patient to treatment is a constant challenge in oncology. Cell resistance to drugs is a determinant of the response to chemotherapy and radiotherapy and its detection may be of clinical relevance. During the last decade, several studies have sought to define the role of expression of transmembrane carriers such as the *MDR-1*, *MRP* and *LRP* genes in the survival from and risk of relapse for childhood acute lymphoblastic leukemia. Some questions about the inter-relationship between drug resistance genes and leukemia are relevant: among the different methods available for the study of resistance, is there one that can be recommended over the others? Is the application of relatively simpler techniques such as RT-PCR comparable to more sophisticated techniques such as biological methods or quantitation by real-time PCR? Among the different transmembrane carrier proteins is there a specific one whose expression may prove to be a prognostic factor?

Some of the relevant studies on transmembrane carrier proteins published over the last few years using different methodologies are presented in Table 4, along with the data obtained. Probably one of the main reasons for the contradictory results from studies on drug resistance relates to methodology. While Dhooge et al.,^12^ using immunohistochemistry, demonstrated strong correlation between p-glycoprotein positivity upon diagnosis and unfavorable clinical course, Wuchter et al.,^14^ in a study on the expression of *MDR-1* using flow cytometry and the rhodamine test, did not detect a correlation between the expression of this gene and worsened overall survival. The use of methodology that will quantify or directly measure gene expression seems to be the most adequate procedure for the definition of the participation of the gene under study in the resistance phenotype. On the other hand, the question becomes even more complex when we consider the molecular aspect of the regulation of the expression of genes such as *MDR-1*. Yague et al.^17^ showed that p-glycoprotein expression in leukemic cells exposed to chemotherapy is regulated by two distinct processes, i.e. the stabilization of messenger RNA and the initiation of the translation process. The *in vitro* study of these phenomena reveals that *MDR-1* overexpression does not always occur via the activation of transcription. Illmer et al.^18^ demonstrated that not only the function but also the polymorphism of the *MDR-1* gene are implicated in the possibility of a response to the treatment of acute myelogenous leukemia (AML). Variant alleles of the *MDR-1* gene influence the protein expression and function of p-glycoprotein, and are possibly related to the pharmacokinetic effects of this protein. These phenomena occurring at the molecular level may partly explain the divergent results that are often reported in different studies involving direct RNA quantitation or the evaluation of protein function.

**Table 4:** Some studies of transmembrane carrier proteins and their respective results

Authors	Methodology	Patients	Results
den Boer et al., 1998^6^	Flow cytometry with monoclonal antibodies	141 children with ALL (112 upon diagnosis and 29 relapses); 27 children with AML	No difference in *MDR-1* and *MRP* expression upon diagnosis and relapse; *LRP* 1.6 times higher in multiple relapses. Co-expression of two or three genes with no impact on survival.
Kanerva et al., 1999^10^	Flow cytometry with monoclonal antibodies	118 children with ALL (103 upon diagnosis and 15 relapses)	No correlation between p-gp expression and the initial response to chemotherapy. Patients with ALL-T have higher p-gp expression (p = 0.002)
Kakihara et al., 1999^11^	Semiquantitative RT-PCR for *MRP* and *LRP*	40 children with ALL upon diagnosis	Absence of correlation between gene expression and age or leukocyte count. No difference in gene expression between the groups at high and low risk of relapse. Absence of impact on survival.
Dhooge et al., 1999^12^	Immunohistochemistry for p-gp in bone marrow	102 children with ALL upon diagnosis and 35 relapses	P-gp positive upon diagnosis – unfavorable course (p = 0.02). P-gp positive upon relapse (34%): twice the risk of unfavorable course (multivariate analysis).
Ogretmen et al., 2000^13^	Semiquantitative RT-PCR for *MDR-1*, *MRP* and *LRP*	14 children with ALL	*MRP* and *LRP* had higher expression in pre-B-ALL. Absence of difference in *MRP* and *LRP* expression upon relapse.
Wuchter et al., 2000^14^	Rhodamine (function). Flow cytometry (expression). Study of p-gp	121 adults with AML and 102 children with ALL	No correlation with ALL immunophenotype, response to induction, relapse and overall survival rate for adults or children.
Sauerbrey et al., 2002^15^	Semiquantitative RT-PCR for *MRP* and *LRP*	58 children with ALL and 28 relapses	No difference in the expression of the two genes upon diagnosis and relapse. The expression of the two genes seems to be correlated (p = 0.001). Expression of *LRP* upon diagnosis: a lower tendency for the patient to remain in first clinical remission.
Plasschaert et al., 2003^16^	Semiquantitative RT-PCR for *MDR-1* and *MRP*, and flow cytometry for rhodamine	36 children and 35 adults with ALL	High p-gp expression in adult ALL-T (p < 0.001). Low correlation between RNA expression and P-gp activity. *MRP* expression has no impact on survival (multivariate analysis). P-gp is correlated with worsened ALL survival only for adults (p = 0.01)

*ALL = acute lymphoblastic leukemia; *MDR-1* = multiple drug resistance-1; *MRP* = multiple drug resistance proteins; *LRP* = pulmonary resistance protein; AML = acute myeloid leukemia; p-gp = p-glycoprotein.*

The percentage positivity for the genes studied that is reported in the literature is widely variable and essentially depends on the study method employed, the positivity cut-off values, and the sampling characteristics of the patients studied. In the present study, 40% of the patients showed increased *MDR-1* expression, 16.6% showed increased *MRP* expression, and 33% showed increased *LRP* expression upon diagnosis. Double positivity for the expression of drug resistance genes was detected in 26.6% of the cases. Gupta et al.^19^ reported levels of *MDR-1* expression ranging from 4 to 60% in acute lymphoblastic leukemia blasts upon diagnosis. Sauerbrey et al.^15^ detected increased *LRP* expression upon diagnosis and relapse in 47% and 68% of children with acute lymphoblastic leukemia, respectively. In our sample, we obtained *LRP* positivity of less than 33.3% upon diagnosis, a fact that may be related to the cutoff level for positivity adopted, or even to characteristics inherent to the population studied. Few data are available on the prevalence of *MRP* expression in childhood acute lymphoblastic leukemia upon diagnosis, and the 16.6% value obtained in the present study may contribute towards the definition of this prevalence. Only one study^6^ has attempted to define the impact of simultaneous expression of two or more resistance genes in the same patient and, in agreement with the data reported in the present work, that study did not detect an effect of the co-expression of resistance genes on event-free survival.

The expression of the *MRP* gene upon diagnosis was not related to a worsening of event-free survival. Sauerbrey et al.^15^ also did not find a correlation between *MRP* expression upon diagnosis and a worsened survival of children with a diagnosis of acute lymphoblastic leukemia. Plasschaert et al.,^16^ in a study on the determination of *MRP* by semiquantitative RT-PCR and flow cytometry, showed that increased *MRP* expression upon diagnosis had no impact on the event-free survival of children or adults. Kakihara et al.^11^ also did not detect an adverse impact from high *MRP* expression upon diagnosis, on the survival of children with acute lymphoblastic leukemia.

In the present study, we observed that increased *LRP* upon diagnosis was strongly related to worsened event-free survival. Patients with positive *LRP* upon diagnosis presented a cumulative survival rate of 25.0% over a period of 48 months, in comparison with an 83.5% cumulative rate for LRP-negative patients (p = 0.005). Sauerbrey et al.^15^ demonstrated that children with a diagnosis of acute lymphoblastic leukemia and high *LRP* expression, as assessed by semiquantitative RT-PCR, showed a lower tendency to remain in first clinical remission, although this finding did not reach statistical significance. den Boer et al.^6^ did not detect an adverse impact from *LRP* expression upon diagnosis, but only observed an increase in *LRP* expression in multiple relapses. Kakihara et al.^11^ also found no correlation between the expression of the *LRP* gene, as determined by RT-PCR, and variables such as age and leukocyte count upon diagnosis, nor did they observe worsened survival in patients with high *LRP* expression upon diagnosis.

The evaluation of the cases of relapse may be of descriptive interest. Case 1 showed minimal difference in the expression profile for the three genes studied, when evaluated upon diagnosis and relapse. This finding contributes to the understanding of resistance to antineoplastic drugs as a multifactorial event in molecular terms, with other factors not considered in the present study possibly contributing directly to the genesis of the resistance phenotype. In case 2, inversion of the expression of resistance genes was found when assessed upon relapse, with absence of positivity for the *MDR-1* gene and high expression of the *MRP* and *LRP* genes (3.92 and 4.57, respectively). These findings raise the hypothesis that the initial *MDR-1*-positive leukemic clone was sensitive to chemotherapy and that a subpopulation with high expression of the *MRP* and *LRP* genes emerged when relapse occurred. In a study on early resistance to treatment in children with acute lymphoblastic leukemia, Brisco et al.^20^ reported that a biphasic decrease in the number of leukemic cells occurred at the beginning of therapy. Treatment induces rapid apoptosis of the sensitive cell fraction, leaving a small, but substantial, number of resistant cells. In this case, it is possible that treatment-resistant cells were present from the time of diagnosis and that their resistance was probably due to genetic or epigenetic events during the proliferation of the leukemic clone.

There are some limitations on the assessment of the data obtained in the present study. The length of time for which patients were observed was short, considering the long-term possibility of the occurrence of a relapse in acute lymphoblastic leukemia. Also, distinct treatment protocols were used (BGTCL 93 and BGTCL 99). These limitations may erroneously express the importance of the protein in question as a mechanism for resistance to a specific chemotherapeutic agent.

## CONCLUSIONS

The study of drug resistance by semi-quantitative RT-PCR may be of clinical relevance. The present study identified resistance genes such as *LRP*, which may be possibly associated with the clinical course of acute lymphoblastic leukemia in children. However, only prospective clinical studies with larger patient series, with a longer time of patient observation and with comparison of the results with techniques of quantification by real time PCR, can validate the participation of the *LRP* gene in the drug resistance phenotype of childhood acute lymphoblastic leukemia.
